# Non-traumatic Thoracic Aortic Dissection in a Healthy Patient

**DOI:** 10.7759/cureus.24826

**Published:** 2022-05-08

**Authors:** Molly S Jain, Enkhmaa Luvsannyam, Keisa Mokenela, Ayola R Leitao, Ayesha E Leitao

**Affiliations:** 1 Medicine, Saint James School of Medicine, Park Ridge, USA; 2 Surgery, Avalon University School of Medicine, Willemstad, CUW; 3 Internal Medicine, Saint James School of Medicine, Park Ridge, USA; 4 Internal Medicine, West Suburban Medical Center, Oak Park, USA

**Keywords:** debakey type 1, stanford type a, cardiovascular, back pain, chest pain, aortic dissection

## Abstract

Aortic dissection (AD) is a serious condition, which typically affects men more than women. Strongly established risk factors include uncontrolled hypertension, connective tissue disorders, advanced age, cocaine use, and aortic injury. We believe it's important to report this case due to the occurrence of Stanford type A AD in a healthy 40-year-old male devoid of genetic, medical, family, and environmental risk factors. This patient presented with a tearing anterior chest pain radiating to the back and was promptly diagnosed and managed with surgical replacement and grafting of the diseased aorta and its involved branches. Therefore, it is imperative to include AD as a differential in atypical case scenarios and case presentations, as missed and delayed diagnosis and management would worsen the clinical outcomes.

## Introduction

Aortic dissection (AD) is characterized as a tear between the intimal and medial layer of the aortic wall [1]. The two subcategories of AD according to the Stanford classification include type A, in which dissection is produced at the level of the ascending aorta, and type B, where dissection occurs along the descending aorta with the ability to extend through the entire length of the vasculature [1]. The DeBakey classification system distinguishes between type I, II, and III dissections. Type I AD involves the entire aorta, type II involves the ascending aorta, and type III involves the descending aorta only [2].

Although relatively uncommon, type A AD is among the most urgent cardiac pathologies. Predispositions to a type A AD include male gender, history of smoking, history of connective tissue disorders, age greater than 60 years, and long-standing uncontrolled hypertension [1]. The ascending aorta is subjected to the highest systolic pressure in the circulatory system; any additional rise in pressure increases the chances of an aneurysm [1]. Classical symptoms associated with AD include sudden onset of severe tearing chest pain radiating to the back. Additionally, loss of consciousness, tachypnea, hypotension, and diminished pulse pressure can also be present. According to von Kodolitsch et al., variables such as abrupt-onset tearing/ripping aortic pain, mediastinal widening, and pulse differential allowed them to identify more than 95% of acute ADs in their study [3]. 

Despite recent technological and pharmacological advancements, diagnosing and managing AD still remains challenging [4]. Computed tomography, magnetic resonance imaging, and echocardiography are commonly used diagnostic tools for aortic pathologies [1]. However, echocardiography remains the technique of choice for the diagnosis of a type A aortic aneurysm due to its use of ultrasound waves to produce images of the heart, aorta, and esophagus [1]. Type A AD is a medical emergency and requires immediate surgical correction. This surgical procedure involves the fortification of the aorta with the use of a synthetic tube. In addition to surgical intervention, the use of medications such as beta-blockers may be used for heart rate and blood pressure control [1]. The pharmacological intervention aims to reduce the chances of dissection exacerbation along with acute life-threatening medical complications such as stroke, organ failure, aortic valve failure, and severe internal bleeding.

We present the case of a healthy 40-year-old male who was suddenly diagnosed with Stanford type A AD. Prompt diagnosis, medical management, absence of postoperative complications, and strict postoperative compliance to medical treatment/follow-ups allowed a favorable prognosis to full recovery in the patient.

## Case presentation

This is a case of a 40-year-old healthy male who presented with a history of tearing chest and back pain. The patient had no significant past medical and surgical history. The patient did not smoke, use illicit drugs, or consume alcohol. The patient worked as a nurse. He had allergic symptoms reported from sulfa medications. The patient denied any significant family history including genetic diseases such as Marfan syndrome, aortic dissection, aortic aneurysm, hypertension, or sudden death.

According to the patient, he experienced sharp chest pain during a visit to the dentist and within a few hours, he felt nauseous and started vomiting. The chest pain continued intermittently for four days and happened usually after exertion. The patient took no medications to aid with the pain and finally decided to seek medical attention. He further presented to the emergency department (ED) on September 7, 2020, with continued chest and back pain for four days. During his visit to ED, he was hemodynamically stable with normal vitals: blood pressure 125/80 mmHg and heart rate 80 bpm. During the initial assessment, the patient reported severe chest pain and rated it 8/10 on scale. Transesophageal echocardiogram (TEE) results were consistent with AD extending to the descending aorta with the flow in both true and false lumen. The patient was immediately taken to the operative room and went through a surgical procedure of right axillary vein grafting and cannulation along with ascending aorta and hemiarch replacement. There were no significant peri or postoperative complications and the patient remained stable post-surgically.

**Figure 1:**
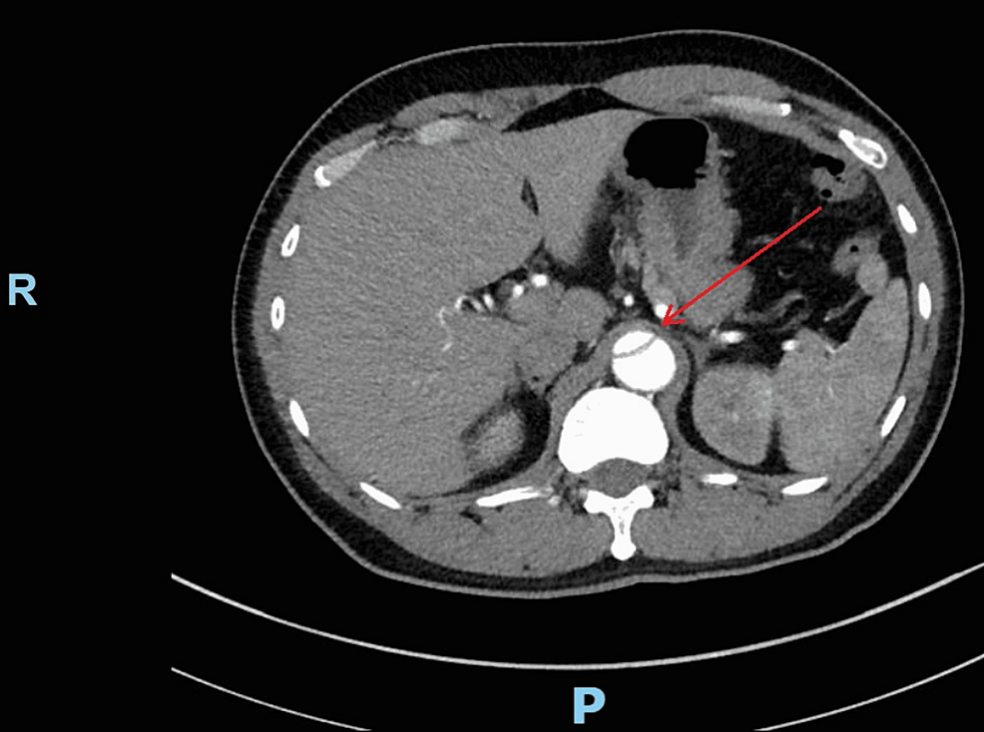
Axial CT angiography of the abdomen with contrast showing aortic dissection

On postoperative day one, the patient had difficulty breathing due to dyspnea and chest pain. Subsequently, the patient required intubation, and his breathing recovered within an hour. He further reported mild tingling in his right hand. His immediate physical assessment was unremarkable. Additionally, the patient also denied visual disturbances, lightheadedness, vomiting, or nausea. The patient remained admitted for seven days and reported no major postoperative complications thereafter. The patient was discharged with the following outpatient medications: amlodipine, acetaminophen, aspirin, carvedilol, hydralazine, magnesium oxide, and tramadol. He was advised to monitor his blood pressure regularly at home and avoid contact sports, heavy lifting, or overexertion. He was also recommended to regularly follow up with the cardiologist and cardiac surgeon. Three months post surgery, a CT angiography was performed, which confirmed DeBakey type I AD beginning at the origin of the right innominate artery extending to the level of the inferior mesenteric artery. All major branches have an origin in the true lumen. No other changes were noted and there was no definitive extension of the dissection into any other branching vessels (Figure 1). 

Currently, the patient is recovering well and reports no chest pain, shortness of breath, palpitations, headache, dizziness, or fever. His vitals have been consistently stable and his blood pressure has been normotensive. The patient is adherent to his regular follow-up visits and medication regimen. He is planning to return back to work once he fully gets clearance from the doctors for his stable health.

## Discussion

Acute thoracic aortic dissection (ATAD) is a serious condition caused by an aortic intimal tear due to a presumed weakened vessel wall [5]. This causes a crack, which allows blood to flow into the medial layer causing separation of the aortic wall layers and creating a false lumen [5]. The morbidity and mortality rates (MR) of ATAD remain high despite improvements in medical diagnosis and management over the years [6]. Due to early high MR, defining a true incidence rate of ATAD is difficult; however, the incidence has doubled in the general population and even more in older individuals in the past decades. Approximately 20-49% of patients with acute AD die before reaching the hospital, and MR can reach 50% within the first 24 hours due to delayed or missed diagnosis [6]. Without treatment, MR rises significantly reaching 3% per hour during the first 24 hours, 30% at week one, and 90% at year one [7]. Approximately one-third of late deaths are caused by complications of AD [7].

AD can lead to life-threatening complications including myocardial ischemia, cardiac tamponade, aortic regurgitation and rupture, end-organ ischemia, shock, and death [8]. Obstruction of the branching arteries may lead to malperfusion syndrome with a consequence of permanent paraplegia [8]. In our case, the patient reported tingling in his right hand postoperatively; however, his physical exam findings were within normal limits and his symptom improved within a few days. Later, his CTA confirmed no extension of the dissection into other branches of the aorta. Surgical treatment is indicated for type A and certain type B ADs; however, the optimal treatment of type B AD remains controversial [8]. Our patient was diagnosed with type A AD with an initial assessment. More specifically, DeBakey type I AD was identified with the flow in both true and false lumen. As a medical emergency, surgery was performed immediately to improve patient morbidity and mortality. After a successful surgical intervention, the patient is then managed medically to stabilize his condition.

A study by Kohl et al. reported that DeBakey type I AD had significantly higher in-hospital MR and malperfusion-related complications compared to type II AD [9]. The reason was explained by the incomplete resection of the entry tear leaving residual dissected arch and descending aorta in type I dissection repair while no residual dissection remains after type II dissection repair. However, the five-year survival rates of both type I and II AD groups were similar overall and among the matched cohorts for patients who survived hospital discharge [9]. Another study by Von Segesser et al. recommended that the proper management of AD should be in accordance with the site of the predominant lesion, and aortic arch replacement through median sternotomy is recommended for patients with an enlarged aortic diameter and aortic insufficiency [10]. In our case, surgical repair was completed successfully as the predominant lesion was found in the ascending aorta and no extension of the dissection was seen. 

The highlight of this case is the presentation of DeBakey type I ATAD in a young, healthy patient. To our knowledge, this is the first case reporting an ascending AD extending into the descending aorta in a patient with no significant risk factor. While acute ADs seen in younger patients are usually a result of genetic conditions such as Marfan syndrome, our patient did not have a family history of any genetic diseases. The patient does not have hypertension, dyslipidemia, atherosclerosis, or any other risk factor contributing to his condition. Moreover, he did not have a history of cardiac or other thoracic surgery that could lead to iatrogenic thoracic AD. A genetic predisposition of thoracic aortic aneurysm and dissection that ran in the family could be the explanation in this case even though no one in the family had displayed the condition prior to the patient. Regardless of patient age and lack of risk factors, surgeons and clinicians must have a high clinical suspicion of acute AD in patients presenting with a tearing chest and back pain. An early evaluation and diagnosis will aid in an appropriate treatment plan and patient survival. Optimal medical management and regular follow-ups are required to improve the patient’s quality of life and prevent serious complications.

## Conclusions

This case signifies the occurrence of a rare condition in a young, healthy patient. It is important to note that regardless of the type, AD is a life-threatening cardiac pathology that requires immediate medical attention to prevent serious complications and even death. It must be considered on top of the differentials when patients present with classic symptoms regardless of patient age, gender, and risk factors. Appropriate physical examination and imaging will aid in prompt diagnosis and management plan. Growing advancements in imaging and pharmacology technologies continue to improve the short and long-term prognosis of patients with acute AD. However, further research is warranted in this area to improve overall mortality.
